# Establishment and Validation of an Integrated Microfluidic Step Emulsification Chip Supporting Droplet Digital Nucleic Acid Analysis

**DOI:** 10.3390/bios13090888

**Published:** 2023-09-18

**Authors:** Gangyin Luo, Ying Zhang, Shun Wang, Xinbei Lv, Tianhang Yang, Jinxian Wang

**Affiliations:** 1 Suzhou Institute of Biomedical Engineering and Technology, Chinese Academy of Sciences, Suzhou 215163, China; luogy@sibet.ac.cn (G.L.); wangs@sibet.ac.cn (S.W.); 2 School of Biomedical Engineering (Suzhou), Division of Life Sciences and Medicine, University of Science and Technology of China, Hefei 230026, China; 3 JiHua Laboratory, Foshan 528251, China; zhangyin@jihualab.ac.cn; 4 Qingdao Innovation and Development Base, Harbin Engineering University, Qingdao 266000, China; lvxinbei@hrbeu.edu.cn

**Keywords:** step emulsification, micro-droplet, integrated microfluidic chip, nucleic acid detection

## Abstract

Uniform and stable droplet generation is critical for accurate and efficient digital nucleic acid analysis (dNAA). In this study, an integrated microfluidic step emulsification device with wide-range droplet generation capability, small device dimensions, convenient fabrication strategy, low contamination and high robustness was developed. A tree-shaped droplet generation nozzle distribution design was proposed to increase the uniformity of droplet generation by equating flow rates, and the flow field in the design was numerically simulated. Theoretical analysis and comparative experiments on droplet size were performed regarding the influences of nozzle dimensions and surface properties. With incubation and hydrophobic reagent treatment, droplets as small as 73.1 μm were generated with multiplex nozzles of 18 μm (h) × 80 μm (w). The droplets were then collected into a standard PCR tube and an on-chip monolayer droplet collection chamber, without manual transfer and sample contamination. The oil-to-sample volume ratio in the PCR tube was recorded during collection. In the end, the droplets generated and collected using the microfluidic device proved to be stable and uniform for nucleic acid amplification and detection. This study provides reliable characteristic information for the design and fabrication of a micro-droplet generation device, and represents a promising approach for the realization of a three-in-one dNAA device under a step emulsification method.

## 1. Introduction

Nucleic acid amplification techniques can increase target nucleic acids into millions of copies in dozens of amplification cycles. It has been widely used in scenarios such as food safety evaluation, forensic identification, clinical diagnosis, and particularly during epidemiological investigation under the coronavirus disease 2019 (COVID-19) pandemic [1,2].

DNAA is a method used for the absolute quantification of nucleic acid molecules. Samples are divided into enormous amounts of micro units with homogeneous volumes, and then every unit undergoes an amplification process. The unit signals are read out based on fluorescence detection, and the ratio of positive droplets to total droplets can be obtained to calculate target nucleic acid concentrations [3].

Based on the insolubility between oil and water, droplet dNAA (ddNAA) is one of the dNAA methods that disperses sample solutions into micro-droplets in an oil phase fluid, using microfluidic approaches such as flow focusing, coaxial flow, step emulsification, ink-jetting, ultrasound excitation, oscillating, etc. [4,5,6,7,8,9]. For a certain volume of sample solution, generating droplets with smaller sizes means larger numbers of droplets would be obtained, which leads to a higher detection sensibility. For a 20 μL sample liquid, typically 20,000–50,000 droplets are generated in the ddNAA process [10]. Therefore, high-throughput droplet generation is significantly essential for investigation applications that require fast test results.

Commercial microfluidic chips using the flow focusing method can disperse 20 μL of sample mixture into 90 μm–120 μm diameter droplets in 1.5 min–5 min, mainly under shearing forces induced by the oil phase’s hydraulic force, but this method usually requires a channel width of less than 80 μm [11]. The fabrication difficulty and cost at such scale are relatively high, regardless of soft lithography or precision machining, especially for lab research and product prototyping applications. For microfluidic chips with larger microchannel scales, small droplets can be obtained by raising the oil-to-sample volume ratio. However, this results in wasted oil phase and poor amplification performance. Moreover, the droplet size is extremely sensitive to the flow rates of the two phases in the flow focusing method and T-junction method, which requires a precise flow rate control group [12,13]. Step emulsification takes advantage of the density difference between the two phases to enhance the Plateau–Rayleigh instability [14], and it is easier for the disperse phase to form droplets under surface tension. Furthermore, the generated droplet diameter is not sensitive to small flow rates until it encounters a sudden rise at a flow rate threshold where the droplet generation principle transitions. Compared to generating droplets with the same diameter by the flow focusing method, larger channel dimensions and easier flow rate control in a step emulsification system can satisfy the same requirement.

Research involving multiple forms of devices has been conducted to help realize ddNAA with a step emulsification method in recent years. In 2017, Ofner fabricated a high throughput chip via glass etching [15]. The chip contains 364 microfluidic channels (step from 20 μm to 120 μm) in a comb-shaped distribution, and the total generation flow rate can reach 25 mL/h. Droplets of 81 μm diameter were generated. The glass chip is reusable for its compatibility with a high-temperature autoclave, but hydrophilic glass is not suitable for nucleic acid solution droplet generation. In 2021, Shi proposed a microfluidic emulsification device that relied on centrifugal force [16]. A microfluidic nozzle (step from 40 μm to 180 μm) fabricated with two-step soft lithography was connected with a sample chamber, and a 2 cm × 2 cm droplet chamber was preloaded with the oil phase. With a centrifuge rotor, the solution for SARS-CoV-2 N gene detection via droplet digital loop-mediated isothermal amplification (ddLAMP) can be dispersed into 130–175 μm diameter droplets. In 2020, Li generated droplets based on an asymmetrical beveled capillary [17]. The droplet diameter was proven to have a linear relationship with the capillary inner diameter. Droplets of deoxyribonucleic acid (DNA) samples were collected and successfully amplified. Furthermore, in 2020, Schulz designed a cartridge with eight emulsification nozzles in a brush-shaped distribution, and the cartridge can fit into a 2 mL standard polymerase chain reaction (PCR) tube [18]. As many as 6 × 10^5^ droplets of a 66 µm diameter were generated by centrifuging the tube in less than 10 min. The droplets were then amplified directly in the 2 mL tube with droplet dPCR (ddPCR) and ddLAMP reagents. Later in 2021, Schlenker, from the same group, proposed a four-plex ddPCR based on a LabDisk [19]. The LabDisk contains 12 ddPCR units, and for each ddPCR unit, 6 µL of emulsification oil and 8 µL of PCR oil were used. Droplets of 82.7 μm can be generated from the single-step emulsification nozzle in the unit, and then amplified and read out within a pyramidal PCR collecting chamber. The droplets were generated under centrifugal force, using a centrifugal device instead of syringe pumps, which is inconvenient for miniaturization and portability. Peng, in 2021, proposed a compact massive microfluidic device that can be integrated into a PCR tube [20]. The PDMS devices, in three shapes, contained 18–24 nozzles in a circumferential distribution, and were fabricated based on two-step lithography. Droplets can be generated continuously without extra oil supply after premium addition in Peng’s design, but the droplets need to be manually transferred to a PCR tube for amplification. Among all of the above research examples, using in-tube cartridges involves contamination processes, such as cartridge removal or droplets pipetting before/after amplification, whereas on-chip droplets storage usually leads to limited droplet amounts or bulk device volumes.

Moreover, most collecting tubes and monolayer droplet collecting chambers are customized, and are incompatible with commercial amplification devices. In developing a step emulsification microfluidic device integrated with droplet generation, droplet amplification and droplet detection (three-in-one device) facilities would be convenient for the realization of low-contamination ddNAA nucleic acid quantification.

To improve the performance of step emulsification devices for nucleic acid analysis applications, this study proposed a novel microfluidic chip that can be manufactured with a convenient and cost-efficient method, and operates with fewer manual transfer processes. The chip mold fabrication requires only one-step lithography, domestic printing and a domestic UV exposure device. The pressure and flow rate imbalances at the nozzles caused by nozzle distribution were studied and optimized, since these imbalances lead to irregular droplet sizes and/or only a few nozzles that can emulsify the sample [21]. Via simulation, the optimized 16-channel tree-shaped nozzle distribution was proven to have a better flow rate distribution. The entire chip design is compact, and the microstructure is compatible with an on-chip collecting chamber and PCR tube. The influences of microfluidic channel dimensions and surface treatments on the sample droplet size were recorded and analyzed. The collected droplets have a wide size range, a good sample-to-oil ratio, and remain stable after amplification. The experimental results prove that the homemade PDMS chip can be used as a highly efficient, reliable and integrated device for digital droplet nucleic acid analysis applications.

## 2. Materials and Methods

### 2.1. Characterization of Droplet Generation in Microchannel

During step emulsification, the water phase samples were separated into microscale monodisperse droplets in the oil phase, under the combined effect of Laplace pressure difference, gravitational forces induced by the density difference of the two phases, inertial forces, surface tension forces and viscous forces, among which the Laplace pressure difference has less influence than the others in our study. From previous research and our preliminary experiments, micro-droplets generation would transform into extra-large droplets generation or jet flow in specific parameter ranges. Dimensionless number Ca (capillary number), which represents the influence of viscous forces over surface tension forces; Bo (Bond number), which represents the influence of gravitational forces over surface tension forces; and We (Weber number), which represents the influence of inertial forces over surface tension forces, are introduced in our study to characterize the droplet generation processes [22,23,24,25]. The dimensionless numbers are defined as follows:

*Ca* = *μ*_dis_*v*/*γ* (1)

*Bo* = (*ρ*_con_ − *ρ*_dis_)G*D*^2^ (2)

*We* = *ρ*_dis_*Dv*^2^/*γ* (3)

where *μ*_dis_, *v*, *γ*, *ρ*_con_, *ρ*_dis_, G and *D* are the viscosity of the continuous phase, the velocity of the dispersed phase, the interfacial tension between the continuous and dispersed phases, the density of the continuous phase, the density of the dispersed phase, the acceleration of gravity and the droplet diameter, respectively.

The critical Ca, Bo and We values are seen as the constraints on the dripping mode to the jetting mode transition for multi-phase flow. It is commonly admitted in previous research that the critical Ca and We are ∼6 × 10^−3^ and ∼10^−2^–10^−1^, respectively [26]. Droplet generation conditions with Ca and We lower than their critical values are considered to be stable, as the surface tension governs the flow. However, the critical value of Bo number varies in different studies. Since buoyancy is involved in this research, we assumed ∼10^−3^–1 to be the critical Bo number [20,27].

### 2.2. Numerical Simulation for Flow Rate in Microchannel

Variations in the flow rates in different nozzles can lead to unevenness in the droplet sizes. It is difficult to measure the flow rate on a microscale level. Therefore, we conducted a laminar flow simulation (COMSOL Multiphysics 5.6) to simulate the flow conditions inside the microfluidic chip. The flow of sample fluid in microfluidic channels is governed by the Navier–Stokes equation and continuity equation:

*ρ*(*μ*·∇) *u* = ∇·(−*p* + *μ*∇ *u*) (4)

*ρ*∇·*u* = 0 (5)

where *ρ*, *u*, *p* and *μ* are the density, fluid velocity, pressure and viscosity, respectively. The mass flow at the inlet was set as 0.2 μL/s, and the pressure at the nozzle outlets was set as atmospheric pressure.

### 2.3. Nucleic Acid Sample and Reagents

Droplet generation oil for probes (BIO-RAD, Hercules, CA, USA) was chosen as the continuous phase in this study. The samples for PCR amplification were the dispersed phase. In a standard PCR sample with a volume of 15 μL, there were 7.5 μL ddPCR supermix for probes (no dUTP, BIO-RAD, Hercules, California, CA, USA), 0.75 μL λ DNA (SD0011, Thermo Fisher Scientific^TM^, Waltham, MA, USA) as the PCR template, 2.25 μL of customized probe and primer (Invitrogen Trading (Shanghai) Co., Ltd., Shanghai, China) and 4.5 μL of water (Sangon, Shanghai, China) (Table S1). The details of the material properties used for the study are listed in Table 1.

### 2.4. Fabrication Process of the Microdevice

The microfluidic chip designed for droplet generation consists three parts: a glass slide spin-coated with PDMS (polydimethylsiloxane, Sylgard^TM^ 184, Dow, Midland, MI, USA) thin film as a substrate; a 3 mm thick PDMS layer with tree-shaped microchannels and an ascending reservoir for step emulsification; and a 1 mm thick PDMS layer as a top layer for sealing and droplet collection. In this study, the nozzles of the microfluidic channels were designed with several different widths for pattern study. The heights of the microchannels are 18 μm, 28 μm, and 38 μm. The width-to-height ratios (w/h) are larger than 3.5 for all the nozzles, in order to achieve better monodispersive generation [28,29,30]. Molds for the microfluidic channel with heights of 18 μm (nozzle widths = 80, 90, 100, 110, 120, 130, 140, 150 μm) and 28 μm (nozzle width = 100, 110, 120, 130, 140, 150, 160, 170 μm) were made with classic one-step lithography with SU8. The mold with a height of 38 μm (nozzle width = 100, 110, 120, 130, 140, 150, 160, 170, 180 μm) was home-made, with photoresistive dry film (Dupont, Wilmington, DE, USA), a high-precision ink-jet film printer and a UV light source, which is convenient for prototyping. The inlet of the tree-shaped microchannel is connected with a syringe pump containing sample solution. The 16 nozzles of the tree-shaped microchannel are connected with a large reservoir. The sample fluid is divided uniformly by four-stage binary divisions of the tree-shaped microchannel. The microfluidic structures were fabricated with soft lithography, and the large reservoir was manually carved out after the PDMS was cured. For on-chip monolayer droplets collection, a 15 mm × 11 mm square collection chamber with a height of 120 μm was added on the top PDMS layer. The height of the chamber should be smaller than 1.5 times the droplet diameter to prevent the droplets from overlapping and inaccurate quantification. Cylinder pillars were added in the chamber to support the chamber ceiling and help fluid spread evenly via surface tension. Four branches linked to the chip outlet were also attached at the end edge of the chamber to help the droplets spread adequately and uniformly in the chamber. The detailed design and dimensions of the soft lithography masks are provided in Figure S1.

**Figure 1:**
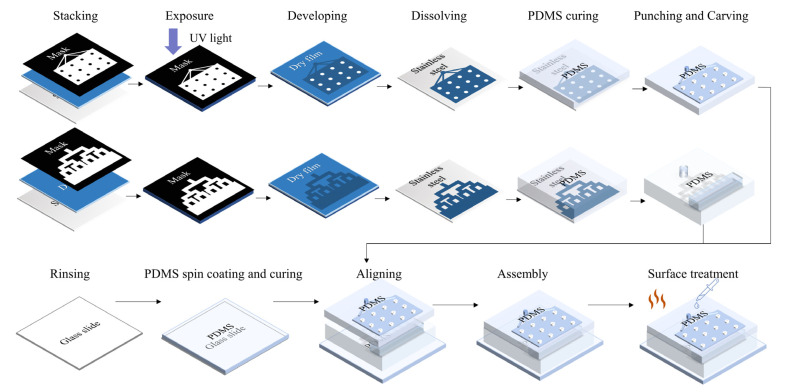
Fabrication of the integrated microfluidic chip.

The three layers were irreversibly bonded together using O_2_ plasma treatment (60 s, 80 W). Small assembled chip dimensions (three-layer integrated chip: 22 mm (l) × 18 mm (w) × 5 mm (h)) and one-step lithography are significant advantages of this study’s design, compared to other emulsification droplet generation devices. Since PDMS becomes hydrophilic after O_2_ plasma treatment, and hydrophily is unfavorable for sample droplet generation, the internal surfaces of the chips were treated with three different methods after bonding which were the following: incubating the chip 48 h at 120 °C; perfusing the channels with hydrophobic reagent (1H, 1H, 2H, 2H -perfluorodecyltriethoxysilane (PFDTES, P122385, Aladdin, Shanghai, China): engineered fluid (3M™ Novec™ 7500, 3M, Saint Paul, MI, USA) (*v*/*v*) = 2%) and maintaining the chip at 120 °C for 5 h; and perfusing the channels with hydrophobic reagent and keeping the chip at 120 °C for 48 h [31]. The complete chip fabrication and treatment process is shown in Figure 1.

### 2.5. Experimental Setup

**Figure 2:**
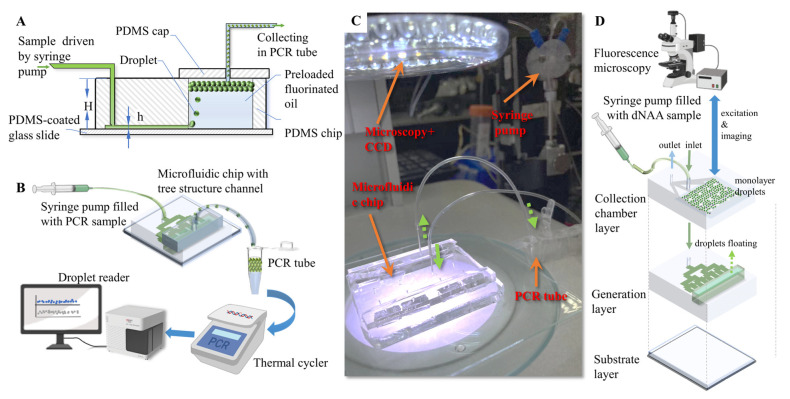
Experimental setup for droplet generation. (**A**) Cross-section illustration of the microfluidic chip. (**B**) Schematic diagram of ddPCR process with microfluidic step emulsification chip. (**C**) Experimental setup. (**D**) Illustration of ddNAA process with integrated three-in-one step emulsification chip.

For all of the characterization experiments in this study, sample solution was pumped into the microfluidic chip with a syringe pump (500 μL ball-end syringe, Tecan, San Jose, CA, USA) at 12 μL/min. PCR oil was preloaded in the reservoir for droplet generation. The sample solutions were pumped through the tree structure microfluidic channel and dispersed from the nozzles into the oil reservoir. Droplets generated from the emulsification were floated into the collection unit, since the amplification reaction solution has a lighter density than the oil phase (Figure 2A). Droplets can be collected either into a standard PCR tube through tubing (Figure 2B,C), or into an on-chip monolayer droplets collecting chamber (Figure 2D). The droplets collected in the standard PCR tube were then amplified in a commercial ddPCR thermal cycling device, and detected in a fluorescence flow cytometry device. The droplet collecting chamber was overlapped on the tree-shaped microchannels so that the 3D microstructure allowed a higher degree of device miniaturization, as illustrated in Figure 2D. An optical upright microscope and a camera (99 fps) were used to record the generation process. Then, the droplets were transferred into a flat monolayer observation chip, and microscopic photos were taken for droplet size statistics. The observation chamber has a height of 120 μm, and droplets with diameters larger than 120 μm were converted as a drum-shaped model to a spherical model for equivalent diameter calculation. Droplets collected in the chamber can be in situ amplified in the future if temperature control units are integrated on the chip substrate and/or materials/reagents are modified. Extra treatments (Figure S2) were also applied on the chip, and the chip containing the liquid was placed in a 60 ℃ incubator for 60 min to demonstrate the feasibility of the chip in amplification with low-temperature reaction reagents. The positive and negative droplets array in the on-chip chamber proved to be distinguishable with fluorescence microscopy (Axio Observer A1, Carl Zeiss AG, Oberkochen, Baden-Wuerttemberg, Germany). However, due to the restrictions of PDMS, only droplet generation and droplet imaging were performed in this study.

### 2.6. Performance Verification of the Collected Droplets

In order to verify the stability and biocompatibility of the microfluidic chip after the fabrication, surface treatment and emulsification processes, droplets were collected and amplified. Droplet amplifications were performed using a commercial PCR thermal cycler (two-step amplification, 40 cycles, Genesy 96E, TIANLONG, Xi’an, Shaanxi, China). FAM channel fluorescence droplet readouts were performed using a droplet reader (DS100 Digital PCR Reader, ZK-Medical, Suzhou, Jiangsu, China) with flow cytometry principles. The target nucleic acid concentration of the sample can be obtained via the Poisson distribution law, with numbers of fluorescence positive droplets and negative droplets [32]. Control group droplets were generated with the same sample solution using a commercial droplet generation device (DS100 Digital PCR Generator, ZK-Medical, Suzhou, Jiangsu, China).

## 3. Results and Discussion

### 3.1. Simulation Results of Nozzle Pressure Distribution

In this study, finite element models were built of the tree-shaped and three classic nozzle distribution designs; each design had 16 nozzles. Their flow rate distributions were simulated and compared. The mass flow at the inlet was set as 0.2 μL/s, and the pressure at the nozzle outlets was set as atmospheric pressure. The cross-sections of all of the nozzles were rectangular (28 μm (h) × 150 μm (w)).

**Figure 3:**
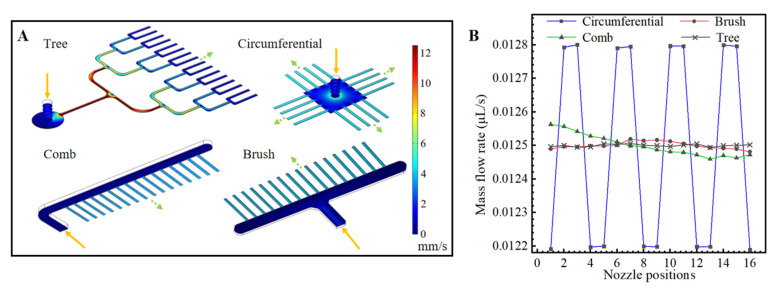
Flow simulation of different chip designs. (**A**) Flow rate distributions in microfluidic structures with four microchannel arrangements. (**B**) Average flow rates at droplet generation nozzles.

The results of the simulations are shown in Figure 3. The mass flow rates are obtained by integrating the sample velocity on the cross-section of the nozzle outlets. Among the tree-shaped distribution, the comb-shaped distribution, the brush-shaped distribution and the circumferential distribution, it could be seen that the circumferential distribution has the largest non-uniformity of flow rates at the nozzles. The tree-shaped distribution has a slightly more uniform distribution than the comb-shaped distribution and the brush-shaped distribution. Therefore, droplets generated with the tree-shape distribution theoretically have better uniformity than the other designs.

### 3.2. Droplet Generation

**Figure 4:**
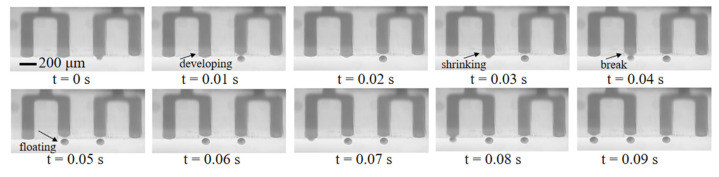
Droplets generation process at four adjacent nozzles.

Multiple droplet generation tests were performed in our microfluidic chips. A dyed sample fluid phase with a slow flow rate (6 μL/min) was used to better observe the droplet emulsification process at the freshly processed hydrophobic nozzles. As shown in Figure 4, the sample liquid was gradually pushed out of the nozzles under hydraulic pressure with increasing upward Laplace force. In a very short time, the sample liquid shrunk at the nozzles, broke at its “neck” under Rayleigh–Plateau instability, formed into a droplet and floated upward due to buoyancy. Droplets with an average diameter of 101.2 μm were generated at two droplets/s at each nozzle (width = 170 μm). Although large flow rates at the nozzles would lead to jetting mode and result in sudden increases in droplet diameter or droplet generation failure, the droplet generation rate can be compensated by increasing the number of channels.

**Figure 5:**
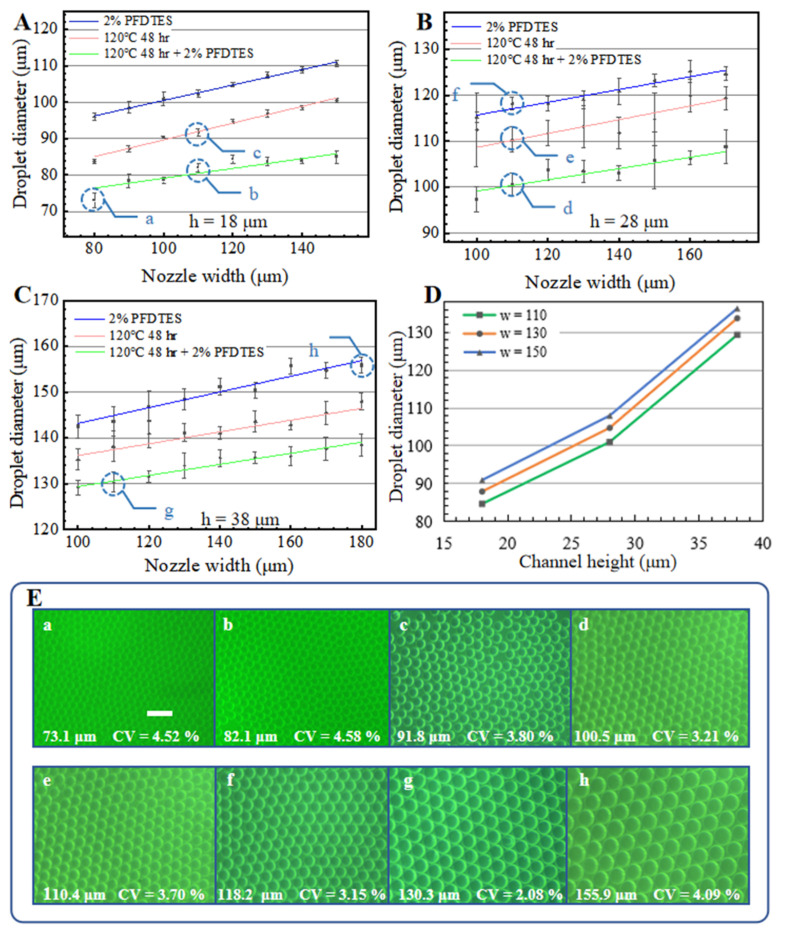
Characterization of droplets generation with the integrated microfluidic chip. Droplets diameter changes with different surface treatments, nozzle widths and nozzle heights (h = 18 μm (**A**), 28 μm (**B**), 38 μm (**C**), respectively). (**D**) shows the increasing droplet diameter with increasing channel height under the same flow rate and same surface treatment method (120 °C 48 h incubation with 2% PFDTES). Error bars represent standard deviations of the droplet diameter. (**E**) The corresponding droplet images of the generation conditions are marked in (**A**–**C**). The scale bar measures 200 μm in (**E**).

Figure 5 shows the characterization results of step emulsification with various nozzle heights, nozzle widths and surface treatment methods. In all of the tested conditions, the Ca is between 2.27 × 10^−4^ and 1.08 × 10^−3^, which is smaller than the critical Ca (∼6 × 10^−3^) in previous studies. The Bo is between 3.97 × 10^−3^ and 1.8 × 10^−2^. The We is between 6.4 × 10^−5^ and 6.8 × 10^−4^. The Bo and We are both smaller than their critical values. Therefore, the droplet generation processes in the tests should be stable in the dripping mode. As shown in Figure 5A–C, the droplet diameter increases linearly with increasing channel width. Figure 5D shows that the droplet diameter also increases with increasing channel height. With the combination of incubation and hydrophobic reagent, the droplet diameter decreases to 77–89% compared to only the hydrophobic reagent treatment, and 84–96% compared to only the incubation treatment, which indicates improvement in the dispersing performance. The decreasing degree is higher with lower channel heights. Figure 5E compares droplets collected in eight typical generation conditions, with an average droplet diameter that ranges from 73.1 μm to 155.9 μm; these results can meet the droplet diameter requirements of most ddNAA applications. The coefficients of variation (CV) are all smaller than 5%, and this proves that the droplet generation processes from the 16 channels are all stable and uniform.

### 3.3. Droplet Collection and Detection

**Figure 6:**
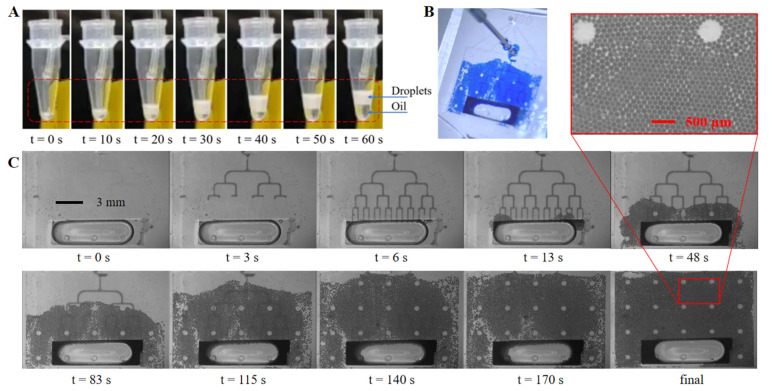
Droplets collection. (**A**) Droplets collection with a standard PCR tube; (**B**) droplets spreading in the on-chip collection chamber; (**C**) droplets generation and collection process with on-chip collection chamber under microscopy.

Figure 6 shows images of the droplet collection tests of two different amplification and detection approaches. In Figure 6A, after being generated at 12 μL/min, the droplets were pumped out of the chip at 24 μL/min and collected in a standard PCR tube through tubing connected with the outlet of the chip. The sample droplets were amplified in the tube, and then detected using fluorescence flow cytometry. It is obvious that the oil-to-sample volume ratio is small (less than one) at the generation stage (1–40 s), compared to a ratio of about two during most flow-focusing ddPCR applications, which indicates less oil phase consumption. Figure 6B shows a picture of the three-layer PDMS chip with on-chip droplet collection, and 6C shows the generation and collection process with dyed droplets filling the on-chip flat collection chamber above the tree-shaped generation structures. The sub-figure in Figure 6C shows details of the droplets distribution as being in a good monolayer array fashion. The on-chip generation and collection processes are shown in Video S1. The 11 mm (l) × 15 mm (w) × 120 μm (h) chamber can contain up to 23,500 droplets of a 90 μm diameter with a total sample volume of 8.9 μL, and 13,200 droplets of a 120 μm diameter with total sample volume of 11.9 μL. While remaining in the same flat chamber, droplets with target nucleic acid can be in situ amplified, then be detected using fluorescence photography and be counted later using image processing. Comparing to previous studies, this integrated collection approach realized in situ amplification and detection in a compact structure, and eliminated manual transfer tasks and possible contamination.

**Figure 7:**
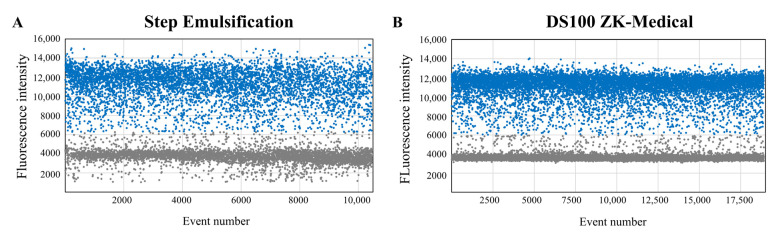
Fluorescence distribution readouts using droplets generated by proposed microfluidic device (**A**), compared with readouts using droplets generated by commercial DS100 (**B**).

Droplets (diameter = 131 μm) containing λDNA templates were generated by the proposed step emulsification device, collected in a standard PCR tube and amplified with the ddPCR method. The control group were droplets (diameter = 128 μm) that were generated by the commercial DS100 Digital PCR Generator. DdPCR amplification and fluorescence readouts for both droplets were completed with the devices mentioned in Section 2.6. Figure 7 shows the droplet fluorescence distributions, where positive droplets (containing amplified target gene) have obviously higher fluorescence intensities than negative droplets (without target gene). Our readout results and the control readout results were both statistically analyzed under the Poisson distribution law to acquire the quantitative nucleic acid concentrations. The concentration results show 614.63 copies/μL with droplets generated by DS100 Digital PCR Generator and 624.22 copies/μL with our device-generated droplets. The results of the two methods are consistent, demonstrating that our integrated chip can realize stable droplet generation for accurate nucleic acid quantitative measurement.

The droplets array in the on-chip chamber can be counted using fluorescence imaging and image stitching. Since the liquid in the chamber will vaporize and leak out during PCR denaturation because PDMS is a porous material, on-chip monolayer amplification was not performed in this study. However, the evaporation can be prevented for 60 min at 60 °C in a chip with extra treatments, which could satisfy the time and temperature requirements of ddRPA and ddLAMP (Figure S2). Multiplex fluorescence of negative and positive droplets can also be detected with pre-amplified droplets injected into the collection chamber (Figure S3), which indicates that the multilayer PDMS structure is compatible with multiplex droplets in situ detection. These results could support the idea that with thermal stable plastic chip and/or low-temperature digital amplification reagents, on-chip in-situ amplification and detection are highly promising with our device.

## 4. Conclusions

Absolute nucleic acid quantification can be realized by dividing nucleic acid samples into individual micro reaction units and analyzing the nucleic acid signal of each unit. Dividing the liquid sample into appropriate volumes with commonly used microfluidic flow-focusing droplet generation methods requires high precision in fabrication and flow control, whereas step emulsification of droplets of similar volumes requires less precision in both. Therefore, this study presented an integrated step emulsification ddNAA microfluidic chip that avoids second-time lithography, regulates the unevenness in the multiplex flow rate, collects droplets with two convenient options and operates with a high degree of convenience, compared to previous step emulsification devices.

In this study, a step emulsification structure with 16 nozzles in a tree-shaped distribution was fabricated simply with one-step lithography. Through flow field simulations, the tree-shaped distribution proved to have a better flow rate distribution compared to the other three distributions. With the microfluidic chip, we then conducted characterization tests and data analysis on the generated droplet diameters. Surface treatments with PFDTES and incubation are helpful for droplet generation by increasing the surface hydrophobicity, which means smaller droplets can be obtained with the same microchannel dimensions. The influences of surface properties, channel height and channel width on droplet sizes were compared in further experiments. Droplets with diameters of 71.3 μm–155.9 μm were generated using microchannels with height of 18, 28 and 38 μm, and widths ranging from 73 μm to180 μm, which could be a reference while choosing an appropriate droplet size for droplet-based nucleic acid detection applications. Two droplet collection methods were proposed in this study. The droplets that were automatically collected in a PCR tube were amplified and successfully read. PCR amplification with on-chip-collected droplets in a monolayer array was restricted because PDMS is gas-permeable, but the three-in-one device was demonstrated to be applicable with low-temperature amplification approaches in the future. Therefore, the research on this interesting step emulsification chip with tree-shaped nozzles and its characterization results are referential for ddNAA applications.

## Figures and Tables

**Table 1:** Material properties used for the study.

*μ*_dis_	*ρ*_con_	*ρ*_dis_	*γ*
1.005 mPa·s	1614 kg/m^3^	998.2 kg/m^3^	8.1 mN/m

## Data Availability

Not applicable.

## Supplementary Materials

The following supporting information can be downloaded at: https://www.mdpi.com/article/10.3390/bios13090888/s1, Table S1: The sequences of primers and probe; Figure S1: Mask film designs and dimensions of the microfluidic layer and the collection chamber; Figure S2: Verification tests of on-chip amplification; Figure S3: Microscopic images of droplets in PDMS–glass chip at fixed position; Video S1: On-chip progress of droplets collection. References [33,34] are cited in the Supplementary Materials.
