# Supplementary material for: Establishment and Validation of an Integrated Microfluidic Step Emulsification Chip Supporting Droplet Digital Nucleic Acid Analysis
Source: Biosensors (Basel). 2023 Sep 18;13(9):888. doi: 10.3390/bios13090888 (PMC10527055; doi:10.3390/bios13090888)
Supplement: Supplementary file 1 [file biosensors-13-00888-s001.zip › biosensors-2486831-supplementary.pdf]

Supporting Information

Establishment and Validation of an Integrated Microfluidic Step Emulsification Chip Supporting Droplet Digital Nucleic Acid Analysis

Gangyin Luo [1,2], Ying Zhang [3], Shun Wang [1], Xinbei Lv [4], Tianhang Yang [1,*] and Jinxian Wang [1,2,*]

Table S1. The sequences of primers and probe [1].

Items	Sequences
Forward primer	CCCAGCAACAGCACAACCC
Reverse primer	GCCGCAGCGTAACTATTACTAATG
Fluorescence probe	6-FAM-ACTGAGCCGTAGCCACTGTCTGTCCT -BHQ1

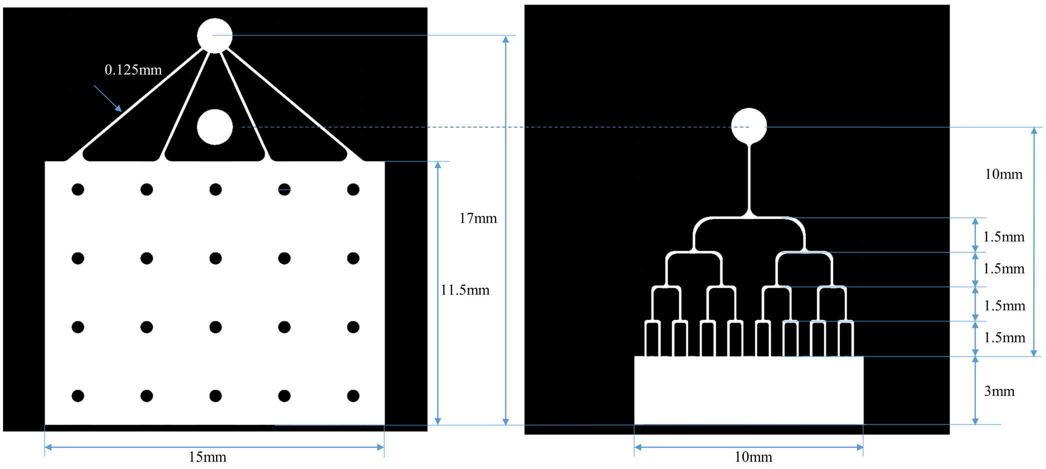

Figure S1. Mask film designs and dimensions of the microfluidic layer and the collection chamber.

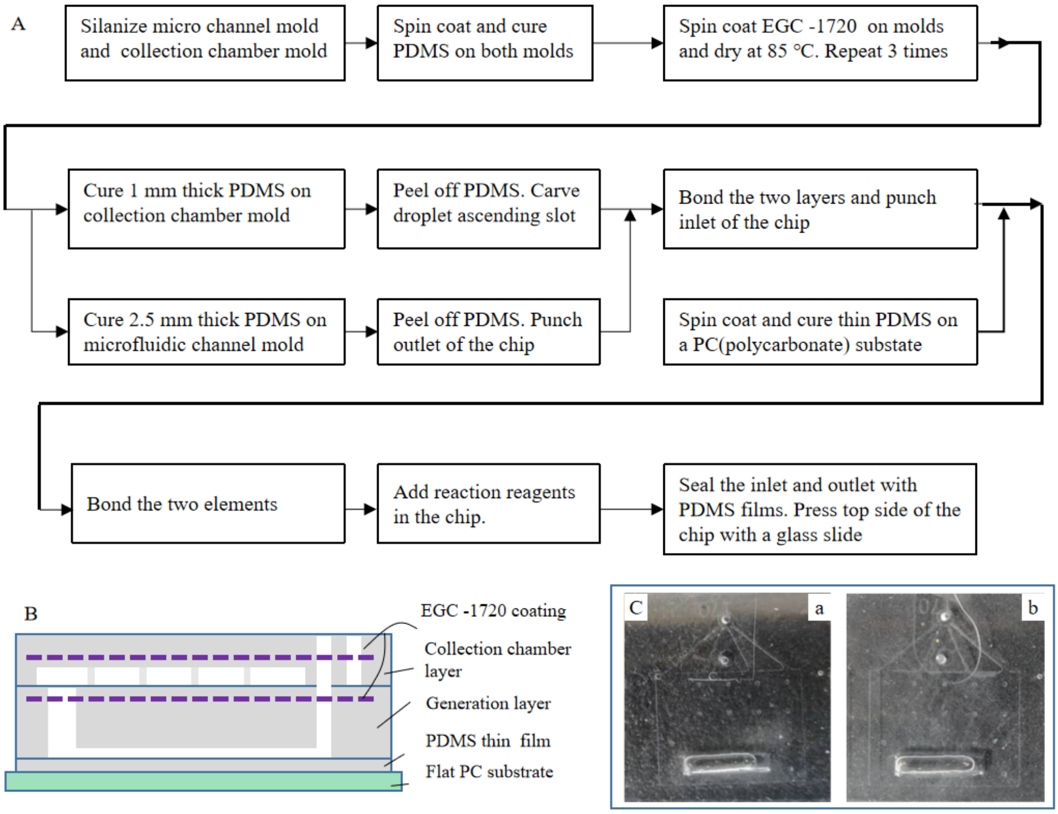

Figure S2. Verification tests of on-chip amplification. A: Processes of modifying PDMS chip for reagents vaporization prevention; B: Multilayer PDMS chip with 3M™ Novec™ 1720 Electronic Grade Coating treatment layers beneath and on top of the monolayer collection chamber [2]; C: Chip sealed with 3M™ Novec™ 7500 Engineered Fluid before(a) and after(b) 60 min in 60 °C incubator.

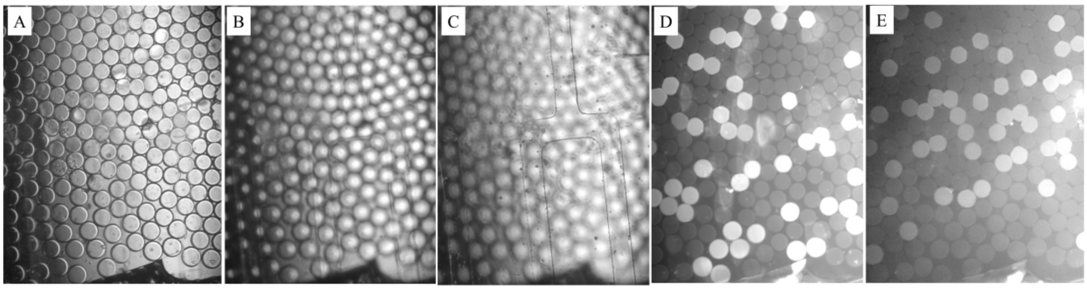

Figure S3. Microscopic gray scale images of droplets in PDMS-glass chip at fixed position. Droplets under bright field focusing on different layers(A-C). A: Image with clear morphology of droplets; B: Image with blur droplets and microchannel; C: Image with clear view of tree-shape microfluidic channels; Blue(D) and red(E) fluorescence images of droplets focusing at the same layer as A.

Video S1. On-chip progress of droplets collection.

References:

1. Bhat, S.; Curach, N.; Mostyn, T.; Bains, G.S.; Griffiths, K.R.; Emslie, K.R. Comparison of methods for accurate quantification of DNA mass concentration with traceability to the international system of units. *Anal. Chem.* **2010**, *82*, 7185–7192.
2. Zhu, Q.; Qiu, L.; Yu, B.; Xu, Y.; Gao, Y.; Pan, T.; Tian, Q.; Song, Q.; Jin, W.; Jin, Q.; et al. Digital PCR on an integrated self-priming compartmentalization chip. *Lab Chip* **2014**, *14*, 1176–1185.
